# Insulin and IGF signaling in the brain: multilevel regulation of synaptic and network homeostasis

**DOI:** 10.3389/fendo.2026.1793536

**Published:** 2026-03-26

**Authors:** LingXi Li, HaiXia He, YuHong Wang

**Affiliations:** 1 Academy of Chinese Medical Sciences, Hunan University of Chinese Medicine, Changsha, China; 2 Hunan Key Laboratory of Traditional Chinese Medicine Prevention & Treatment of Depressive Diseases, Changsha, China; 3 The First Hospital of Hunan University of Chinese Medicine, Changsha, Hunan, China

**Keywords:** diabetic encephalopathy, Hebbian plasticity, insulin and IGF signaling, microenvironment, synaptic homeostatic plasticity

## Abstract

Insulin and insulin-like growth factor (IGF) signaling function as central regulators of synaptic homeostasis, bridging cellular metabolism with plasticity across molecular, synaptic, and network levels. Insulin signaling, is characterized as rapid and transient, and regulates energy metabolism, receptor trafficking, and short-term plasticity, whereas IGF signaling, with more sustained activity, supports neuronal survival, dendritic growth, and long-term remodeling. Together, they contribute to synaptic homeostasis through AMPAR/NMDAR modulation, astrocytic coupling, and activity-dependent scaling. Disruption of these regulatory processes has been associated with aberrant plasticity and altered network stability in Alzheimer’s disease, epilepsy, and diabetic encephalopathy, highlighting their pathophysiological significance. This review synthesizes recent evidence to propose an integrative framework in which insulin and IGF signaling acts as a molecular hub linking metabolic state to synaptic homeostasis. Understanding this cross-scale regulation not only clarifies how metabolic disturbances lead to cognitive decline but also establishes a foundation for novel therapeutic strategies aimed at restoring neural network function in metabolic and neurodegenerative disorders.

## 1 Introduction

Diabetic encephalopathy (DE) is a chronic complication of diabetes affecting the central nervous system and is increasing in prevalence worldwide (1–3). Epidemiological evidence indicates that diabetes nearly doubles the risk of dementia, and the severity of diabetic complications correlates strongly with cognitive impairment (4, 5). These findings highlight an urgent need to clarify the mechanisms linking metabolic dysfunction to brain pathology.

Synaptic plasticity is the cellular substrate of learning and memory and operates across multiple scales, from activity-dependent modifications at individual synapses to homeostatic regulation of network excitability. Beyond cognition, these adaptive processes influence emotional regulation (6), pain perception (7), and sleep (8). Such plasticity depends on coordinated support from the synaptic microenvironment, including extracellular matrix remodeling and glial-mediated metabolic and inflammatory control (9). Owing to its high energetic demand, synaptic stability is particularly vulnerable to metabolic disturbance. In DE, central insulin signaling dysfunction disrupts both activity-dependent and homeostatic plasticity, linking metabolic imbalance to circuit instability.

Although synaptic research has traditionally emphasized Hebbian mechanisms such as long-term potentiation (LTP), long-term depression (LTD), these processes require compensatory mechanisms to prevent network destabilization. Homeostatic plasticity—including synaptic scaling and metaplasticity—maintains firing rates and excitatory–inhibitory balance. Within this framework, insulin and IGF signaling act as metabolic integrators of both Hebbian and homeostatic processes. Beyond modulating LTP and LTD, these pathways regulate synaptic scaling, α-amino-3-hydroxy-5-methyl-4-isoxazolepropionic acid receptors (AMPARs) trafficking, and mitochondrial calcium dynamics, thereby coupling energy metabolism to circuit stability.

Insulin, Insulin-like growth factor 1(IGF-1), and Insulin-like growth factor 2(IGF-2) exert their effects through overlapping receptor systems (IR, IGF-1R, IGF-2R/M6P) that activate PI3K–Akt and MAPK pathways to support synaptic remodeling, neuronal survival, and metabolic coordination (10–12). In diabetes, reduced insulin/IGF availability and signaling impairment promote tau dysregulation, impaired Amyloid-beta(Aβ) clearance, neurotransmitter imbalance, and neuroinflammation, ultimately leading to synaptic dysfunction and neurodegeneration (13, 14).

**Figure 1:**
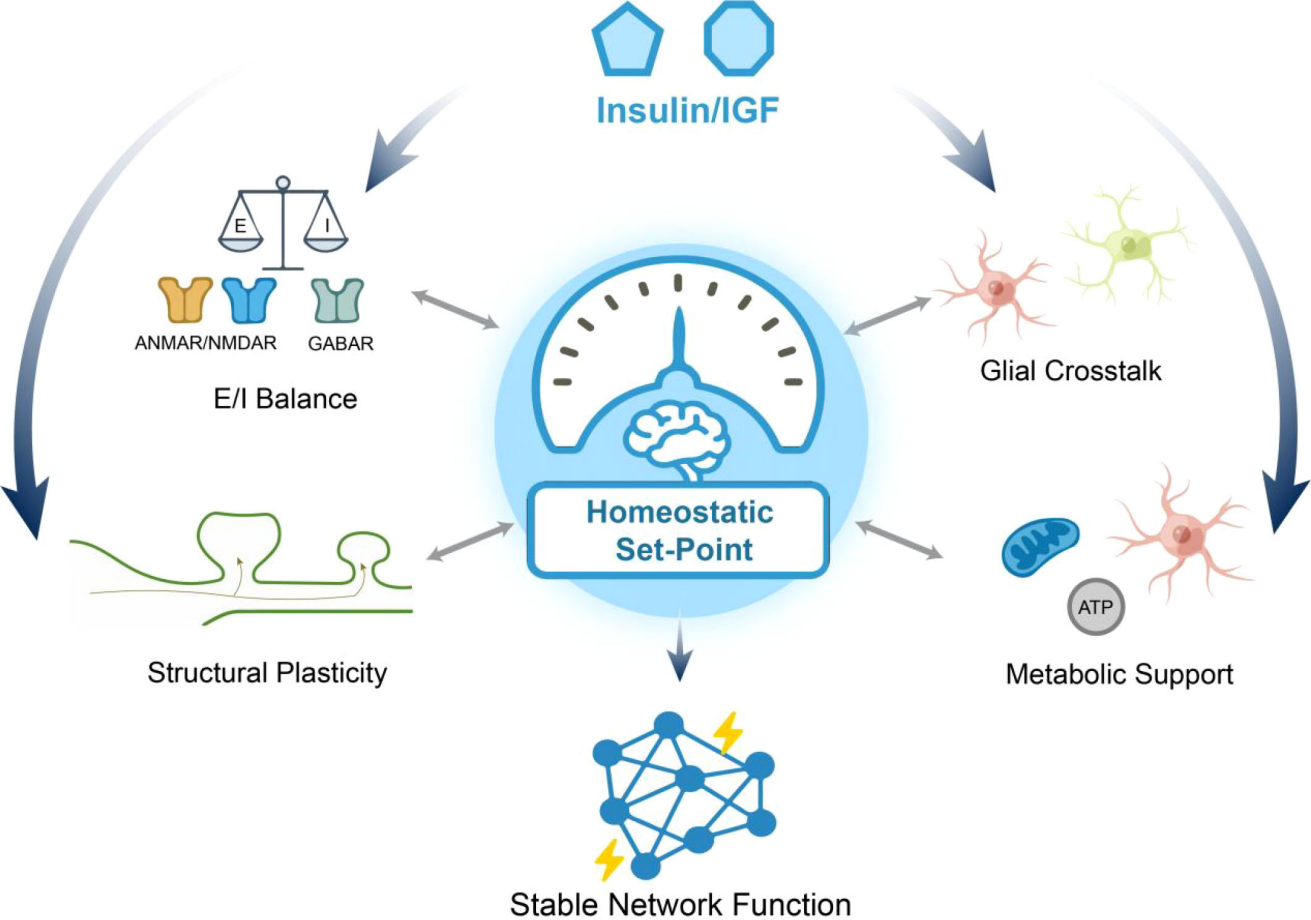
Insulin and IGF signaling: an integrative hub for synaptic and network homeostasis. This schematic model depicts insulin and IGF as a central integrative system that coordinates four pivotal dimensions of neural circuit function to maintain homeostasis. The core Homeostatic Set-Point symbolizes the optimal and stable operating state of the neural network.

This review proposes an integrative framework in which insulin and IGF signaling act as a central hub, coordinating multiple dimensions of synaptic function to maintain network homeostasis. The following sections elaborate on the evidence for insulin and IGF regulation within each dimension outlined in **Figure 1**, beginning with their modulation of basal synaptic transmission. Understanding these mechanisms provides a basis for elucidating how signaling deficits drive cognitive decline in metabolic and neurodegenerative disorders, and for evaluating Insulin and IGF pathways as potential therapeutic targets.

## 2 Insulin and IGF signaling in the regulation of excitation–inhibition balance

Insulin and insulin-like growth factors act through structurally related yet functionally distinct receptors, including the IR, IGF-1R, and IGF-2R. IR and IGF-1R are tyrosine kinase receptors with overlapping downstream cascades but distinct ligand affinities: IR preferentially binds insulin, whereas IGF-1R has higher affinity for IGF-1 and partial affinity for IGF-2 (15). Hybrid IR/IGF-1R complexes further diversify signaling outputs. In contrast, IGF-2R lacks intrinsic kinase activity and primarily regulates IGF-2 trafficking and protein turnover, thereby influencing synaptic proteostasis rather than rapid signal transduction (16). Developmentally, IGF-2 and IGF-2R are enriched during embryonic and early postnatal stages, consistent with roles in circuit maturation, whereas IR and IGF-1R persist into adulthood to support activity-dependent plasticity and metabolic integration (16, 17). The molecular and spatial specificity of insulin and IGF receptors in the brain are shown in **Table 1**.

**Table 1:** Molecular and spatial specificity of insulin and IGF receptors in the brain.

Receptor	Primary ligand	Signaling capacity	Major brain distribution	Developmental profile	References
IR	Insulin > IGF-2	Tyrosine kinase (PI3K–Akt, MAPK)	Hippocampus (CA1, DG), cortex (layers II/III, V), hypothalamus, olfactory bulb; neurons > glia	Present throughout life; peak during perinatal synaptogenesis and adulthood; declines in aged hippocampus	(18–20)
IGF-1R	IGF-1 > IGF-2	Tyrosine kinase (PI3K–Akt–mTOR)	Hippocampus, cortex, cerebellum, hypothalamus, striatum; neurons and astrocytes (low), microglia	Constitutive expression from embryonic to adult; stable with age; upregulated after injury	(21–23)
IGF-2R/M6P	IGF-2	Non-kinase; trafficking/clearance	Choroid plexus, meninges, glial cells (astrocytes, microglia), vascular endothelium; low in neurons	High in embryonic and early postnatal; declines postnatally but re-induced in injury/disease	(24, 25)

Functionally, this receptor diversity translates into distinct yet coordinated control of synaptic efficacy. Through receptor-specific modulation of AMPA and N-methyl-D-aspartate receptor(NMDAR) trafficking, cytoskeletal remodeling, and mitochondrial support, insulin and IGF signaling calibrate excitatory and inhibitory transmission. By linking rapid Hebbian modifications with slower homeostatic scaling mechanisms, these pathways stabilize network excitability under both physiological and pathological conditions.

### 2.1 Bidirectional and decoupled regulation of glutamatergic transmission

The regulation of glutamatergic transmission by insulin and IGF signalling exhibits a refined bidirectional nature. Presynaptically, insulin binding to IR increases the number of vesicles in the readily releasable pool of hippocampal neurons, thereby facilitating glutamate release, an effect reflected in changes in paired-pulse plasticity and an increase in the frequency of miniature excitatory postsynaptic currents (mEPSCs) (26). IGF-1 specifically increases the frequency of spontaneous EPSCs (sEPSCs) through activation of the MAPK/ERK1/2 signalling pathway (27). Notably, this global modulation of release probability may itself constitute a presynaptic scaling mechanism that stabilizes baseline excitatory drive (28).

Postsynaptically, the regulation is more complex and exhibits “decoupled” characteristics. On one hand, the signalling potently enhances NMDAR function: insulin promotes its trafficking to the plasma membrane via SNARE-dependent exocytosis (29) and stimulates tyrosine phosphorylation of NR2A and NR2B subunits via the PI3K/AKT pathway to enhance channel activity and calcium influx (30, 31). IGF-1 enhances field excitatory postsynaptic potentials (fEPSPs) in the CA1 region of the hippocampus in both young and aged rats (32, 33) and rescues excitatory synaptic deficits in models of Pitt-Hopkins syndrome and opioid use disorder by upregulating synaptic NMDARs and AMPARs (34, 35). On the other hand, the same signalling pathways can induce internalization of AMPARs: insulin application reduces the frequency of AMPAR-mediated mEPSCs in visual neurons of the toad (36) and induces hippocampal LTD, suppressing glutamatergic synaptic transmission via tyrosine phosphorylation of the GluA2 subunit and upregulation of the cytoskeletal protein Arc (37–40). In the prefrontal cortex of adult mice, IGF-1 triggers AMPAR endocytosis by phosphorylating mGluR1 via the ERK1/2 pathway, ultimately reducing the amplitude of evoked EPSCs(eEPSCs) (41).

This apparent dissociation between NMDAR enhancement and AMPAR inhibition represents a sophisticated homeostatic strategy. By augmenting NMDAR function, the system facilitates synaptic potentiation and lowers the threshold for plasticity, thereby supporting learning. Concurrently, the regulation of AMPAR membrane trafficking provides precise control over net excitatory gain, preventing runaway network excitation. This dual mechanism effectively integrates Hebbian plasticity with homeostatic metaplasticity, ensuring network stability while preserving the capacity for information storage (42).

### 2.2 Multi-level Enhancement of GABAergic Inhibition

Complementing their regulation of excitatory transmission, insulin and IGF-1 signaling potentiate GABAergic inhibition at multiple synaptic and cellular levels. A principal mechanism involves the modulation of postsynaptic GABAA receptors (GABAARs). Insulin activates PI3K signaling, promoting the association of the p85 subunit’s SH2 domain with GABAARs and phosphorylation of the β2 subunit, which collectively enhance receptor membrane insertion (43–45). This underlies the observed insulin-induced increase in miniature inhibitory postsynaptic current (mIPSC) amplitude, demonstrated both in cultured neurons (46) and within key brain regions including the hippocampus, prefrontal cortex, and amygdala (43, 47–49). In cerebellar granule cells, insulin further elevates inhibitory synapse density by increasing cytoplasmic reactive oxygen species (ROS) to recruit α6 subunit-containing GABAARs (50). IGF-1 exerts complementary actions, such as modulating inhibitory synaptic function in hypothalamic neurons (51) and rescuing Aβ-induced suppression of spontaneous IPSCs in hippocampal neurons (52).

Regulatory control extends to presynaptic and network levels. Insulin facilitates GABA release in a region-specific manner, exhibiting greater potency in the dorsal compared to the ventral dentate gyrus (47). Importantly, insulin can directly modulate the intrinsic excitability of inhibitory interneurons, for instance by lowering the action potential threshold of fast-spiking GABAergic neurons (FSNs), thereby increasing their low-frequency firing (53). This coordinated “up-scaling” of inhibition—spanning postsynaptic receptor efficacy, presynaptic release probability, and interneuron excitability—constitutes a robust inhibitory feedback mechanism. In concert with the bidirectional regulation of excitatory transmission, it positions Insulin and IGF-1 signaling as a master regulator capable of dynamically and precisely calibrating the global excitatory/inhibitory (E/I) balance within neural circuits. This fine-tuning is fundamental for stabilizing network activity and maintaining mean firing rates within a physiological range (51, 53).

### 2.3 Homeostatic tuning of intrinsic excitability via ion channel modulation

Beyond modulating synaptic transmission, insulin and IGF-1 signaling directly regulate neuronal intrinsic excitability by targeting ion channels, establishing a third pillar of homeostatic control. Their effects on potassium channels are notably diverse: IGF-1 inhibits K^+^ channels via MARK signaling in brainstem dorsal column nucleus neurons, delaying repolarization and thereby enhancing EPSCs (54). Conversely, insulin exerts an anti-epileptic effect by activating large-conductance calcium-activated potassium (BK) channels via the Ras–Raf–MAPK pathway, thereby reducing hippocampal hyperexcitability (55, 56). Insulin also suppresses spontaneous oscillatory activity in hypothalamic POMC and NPY/AgRP neurons by activating ATP-sensitive potassium (KATP) channels (57, 58).

The modulation of calcium signaling and neuronal afterhyperpolarization (AHP) is equally critical. Insulin reduces AHP by inhibiting voltage-gated calcium channels (VGCCs) and ryanodine receptor (RyR)-mediated calcium-induced calcium release (CICR) from the endoplasmic reticulum (59). In hippocampal CA1 neurons, this effect involves PI3K- or tyrosine kinase-mediated inhibition of L-type calcium channels (60, 61). IGF-1 facilitates fear extinction learning by reducing medium and slow AHP (mAHP/sAHP) in layer 5 pyramidal neurons of the prelimbic cortex, consequently increasing their firing frequency (62). Additionally, insulin upregulates the membrane expression of TRPV family receptors in human neuroblastoma cells via PI3K/PKC signaling (63). Modulating ion channels provides a direct means of setting the intrinsic threshold for neuronal “output” (action potential generation), independent of synaptic “input” regulation. This form of homeostatic plasticity serves as a fundamental safeguard and ultimate line of defense, ensuring that overall network activity remains within a physiological range during sustained shifts in synaptic strength or under pathological conditions.

## 3 Synaptic efficacy regulation by insulin and IGF signaling

Insulin and IGF signaling are established as master regulators of synaptic efficacy, integrating fast activity-dependent (Hebbian) plasticity with long-term homeostatic adjustments. This coordination stabilizes neural circuits by bidirectionally tuning synaptic strength and maintaining the structural and metabolic integrity of synapses.

### 3.1 Functional and adaptive regulation: integrating Hebbian and homeostatic plasticity

Synaptic plasticity encompasses both Hebbian and homeostatic forms. LTP, a persistent strengthening of synapses following high-frequency stimulation, and LTD, an activity-dependent weakening of synaptic efficacy, represent the two principal forms of Hebbian plasticity. High-frequency stimulation induces LTP through NMDA receptor–dependent calcium influx, triggering CaMKII activation and activity-dependent insertion of AMPA receptors into the postsynaptic membrane. In contrast, low-frequency stimulation promotes LTD via reduced calcium signaling and AMPAR internalization. These activity-dependent processes modify synaptic weight in an input-specific manner and are further refined by spike-timing–dependent plasticity (STDP), in which the temporal order of pre- and postsynaptic firing determines potentiation or depression. However, due to their positive-feedback nature, Hebbian mechanisms can destabilize network activity if left unchecked. Homeostatic plasticity therefore provides a compensatory counterbalance by globally scaling synaptic efficacy and adjusting intrinsic excitability. Insulin and IGF signaling are pivotal to this equilibrium, functioning as bidirectional regulators that integrate Hebbian plasticity with homeostatic mechanisms.

Bidirectional control of AMPARs serves as a central mechanism. LTD and homeostatic downscaling are facilitated by insulin via mTOR- and PI3K/PKC-dependent internalization of GluR2, whereas IGF-1 can promote AMPAR endocytosis through the ERK/mGluR1 pathway (37, 41, 64–66). This insulin-driven AMPAR removal under conditions of elevated activity has been reinterpreted as synaptic downscaling, a hallmark of homeostatic plasticity that prevents pathological hyperexcitability (67). Conversely, LTP is supported by insulin-mediated enhancement of GluR1 phosphorylation and surface expression via protein kinase M zeta (68, 69). In parallel, NMDAR function is potentiated by insulin through PI3K–Akt-dependent tyrosine phosphorylation of NR2 subunits, lowering the threshold for Hebbian plasticity (29–31, 70, 71). This differential regulation is essential for metaplasticity, as evidenced by dose-dependent IGF-1 effects on LTP/LTD thresholds (72) and input-specific modulation via local IGF-1/IGF-2 autocrine signaling in dendritic spines (73). Consequently, the E/I balance is dynamically calibrated, ensuring Hebbian modifications occur within a stabilized network framework that prevents pathological hyperexcitability (67). Collectively, insulin and IGF-1 signaling exert a bidirectional and partially dissociable regulation: AMPAR trafficking provides the substrate for both synaptic potentiation and receptor removal, while NMDAR modulation adjusts the threshold for activity-dependent plasticity. These dual actions establish insulin and IGF as bidirectional regulators that integrate Hebbian plasticity with homeostatic mechanisms, thereby ensuring both adaptability and stability of neural circuits.

### 3.2 Structural and microenvironmental regulation: a cross-scale homeostatic system

Beyond receptor kinetics, synaptic efficacy is governed by a multi-tiered homeostatic system coordinating structural integrity and microenvironmental support. Structural plasticity is directly regulated through cytoskeletal remodeling. Synaptogenesis and spine morphogenesis are promoted via the PI3K/Akt/mTOR–Rac1 axis, with key adaptors like IRSp53 linking signaling to actin dynamics (74–78). This control is functionally restorative, as demonstrated by the rescue of spine loss in models of Alzheimer’s disease (3xTg-AD mice), aging, Methyl-CpG-binding protein 2(MeCP2) mutation, and Pitt-Hopkins syndrome through insulin or IGF-1/IGF-2 administration (35, 79–81).

Importantly, insulin and IGF signaling also operate during early brain development, extending their influence beyond mature synaptic regulation. IGF-2 is highly expressed during embryonic and early postnatal stages, where it regulates progenitor proliferation, neuronal differentiation, and cortical layer formation (82). IGF-1 supports dendritic growth and activity-dependent synaptic refinement (83), while insulin signaling contributes to neuronal survival and metabolic programming of developing circuits (84). Disruption of these pathways during critical developmental windows alters spine patterning, cytoskeletal organization, and long-range connectivity, predisposing neural networks to later instability (27). Moreover, maternal metabolic disturbances can reshape the fetal insulin/IGF axis, influencing microglial maturation and synaptic pruning trajectories, thereby establishing persistent circuit vulnerabilities (85, 86).

The synaptic microenvironment is actively maintained through triad support. First, astrocytic glutamate clearance is enhanced via PI3K-dependent GLT-1 upregulation, while gliotransmission (e.g., ATP/adenosine) is modulated to couple metabolic support to synaptic plasticity (87–90). Second, a critical immune feedback loop with microglia is maintained; neuroinflammation is suppressed via inhibition of the HMGB1/TLR4/NF-κB axis, whereas pro-inflammatory cytokines like TNF-α can induce neuronal insulin resistance, creating a pathological cycle (91–93). Third, energetic and redox homeostasis is orchestrated at mitochondria. IGF-1 optimizes presynaptic calcium buffering and ATP production, whereas IGF-2 receptor activation protects against oxidative stress, preserving synaptic protein integrity (94–96).

Emerging evidence positions insulin signaling as a critical “metabolic checkpoint” regulating microglial synaptic pruning, a key process within the synaptic microenvironment. In microglia-specific insulin receptor knockout (MG-IRKO) models, compensatory shifts toward glycolysis occur, coinciding with impaired phagocytic functions such as Aβ clearance (86). This indicates that intact insulin signaling normally supports oxidative phosphorylation to generate sufficient ATP for synaptic engulfment, with its loss triggering a pathogenic metabolic shift. Further supporting this metabolism-function coupling, inhibition of hexokinase 2 (HK2) was found to enhance fatty acid oxidation via lipoprotein lipase (LPL), increase ATP production, and restore phagocytic capacity (97).

Collectively, Insulin and IGF signaling integrates molecular receptor trafficking, cellular structural changes, and inter-cellular glial dialogue into a coherent, cross-scale homeostatic system. Its disruption impairs function at every level, underscoring its fundamental role in synaptic stability and its relevance as a therapeutic target for neurodegenerative and metabolic brain disorders.

## 4 Maintenance of synaptic homeostatic plasticity by insulin and IGF signaling

Homeostatic plasticity is a fundamental “negative feedback” mechanism that adapts the brain to long-term environmental changes, ensuring neuronal firing rates remain within a physiological dynamic range (98). In contrast to the unidirectional changes driven by Hebbian plasticity, homeostatic mechanisms safeguard overall network stability by bidirectionally adjusting intrinsic excitability and synaptic strength, thus serving as an essential complement. Currently, a substantial body of literature indicates that pharmacological agents, including lithium (99), ketamine (100), IL-33 (101) and retinoic acid (102), target synaptic homeostatic plasticity to address neuropsychiatric disorders.

Within this critical paradigm, insulin and IGF-1 signaling emerge as key hierarchical regulators, operating from molecules to synapses and networks with the core feature of bidirectional regulation and dynamic balance. At the molecular and synaptic level, insulin activates the PI3K–Akt pathway to enhance NMDAR currents, supporting LTP (71, 103), while also inducing AMPAR endocytosis to drive LTD, thus providing flexible, bidirectional control of excitatory transmission (104). AMPARs are identified as a key target for maintaining synaptic homeostatic plasticity. This role is confirmed by IGF-1, which increases the number of AMPARs and GluA1 subunits at the synapse to rescue excitability deficits in various disease models (32–35). IGF-1 also engages the ERK/mGluR1 pathway to induce AMPAR internalization with longer-lasting kinetics and promotes dendritic growth and long-term structural plasticity (41). This bidirectional regulation extends to intrinsic neuronal excitability, where insulin reduces afterhyperpolarization (AHP) currents via calcium channel modulation (59) and activates BK channels to suppress pathological hyperexcitability, as observed in epilepsy models (55, 56). Furthermore, insulin mediates essential metabolic–synaptic coupling by enhancing glucose uptake in both neurons and astrocytes. When impaired, this leads to reduced astrocytic metabolism and glutamate clearance, aggravating network hyperexcitability. Both pathways are shaped by activity-dependent feedback, forming a self-regulating system.

A central function of Insulin and IGF-1 signaling in homeostatic plasticity is the precise calibration of the network-wide mean firing rate (MFR), a critical feedback variable. Emerging evidence emphasizes that IGF-1 helps maintain MFR within a physiological window by regulating presynaptic mitochondrial calcium buffering and ATP release, thereby fine-tuning presynaptic glutamate release and constraining short-term excitability (94). This dual action ensures the preservation of basal transmission while preventing runaway excitation. Importantly, deletion of IGF-1R disrupts presynaptic coupling to the mitochondrial calcium uniporter complex (MCUc), impairing mitochondrial Ca²^+^ uptake and limiting the network’s ability to compensate MFR under hypoactive conditions, underscoring its essential role in loop-level homeostasis (94).

At the systems level, advanced methodologies are crucial for investigating how these pathways stabilize large-scale networks. Multi-electrode array (MEA) platforms, capable of long-term, high-throughput recordings, permit simultaneous monitoring of MFR, burst dynamics, and network synchronization across neuronal ensembles. These approaches are particularly suited to capture how insulin and IGF-1 signaling stabilize excitatory–inhibitory balance, with recent high-density MEAs enabling spatially resolved circuit-level analyses. Complementary *in vivo* insights are provided by fiber photometry, which utilizes genetically encoded sensors (e.g., GCaMP, dLight) to monitor neurochemical and calcium dynamics in freely moving animals. This technique links insulin- and IGF-1-dependent signaling events to real-time network adaptations and, when combined with optogenetic manipulations, allows for causal interrogation of these pathways. Clinically, the network-modulating role of these pathways is supported by findings that intranasal insulin enhances hippocampal–default mode network (DMN) connectivity in individuals with type 2 diabetes, an effect associated with memory improvement (105).

**Figure 2:**
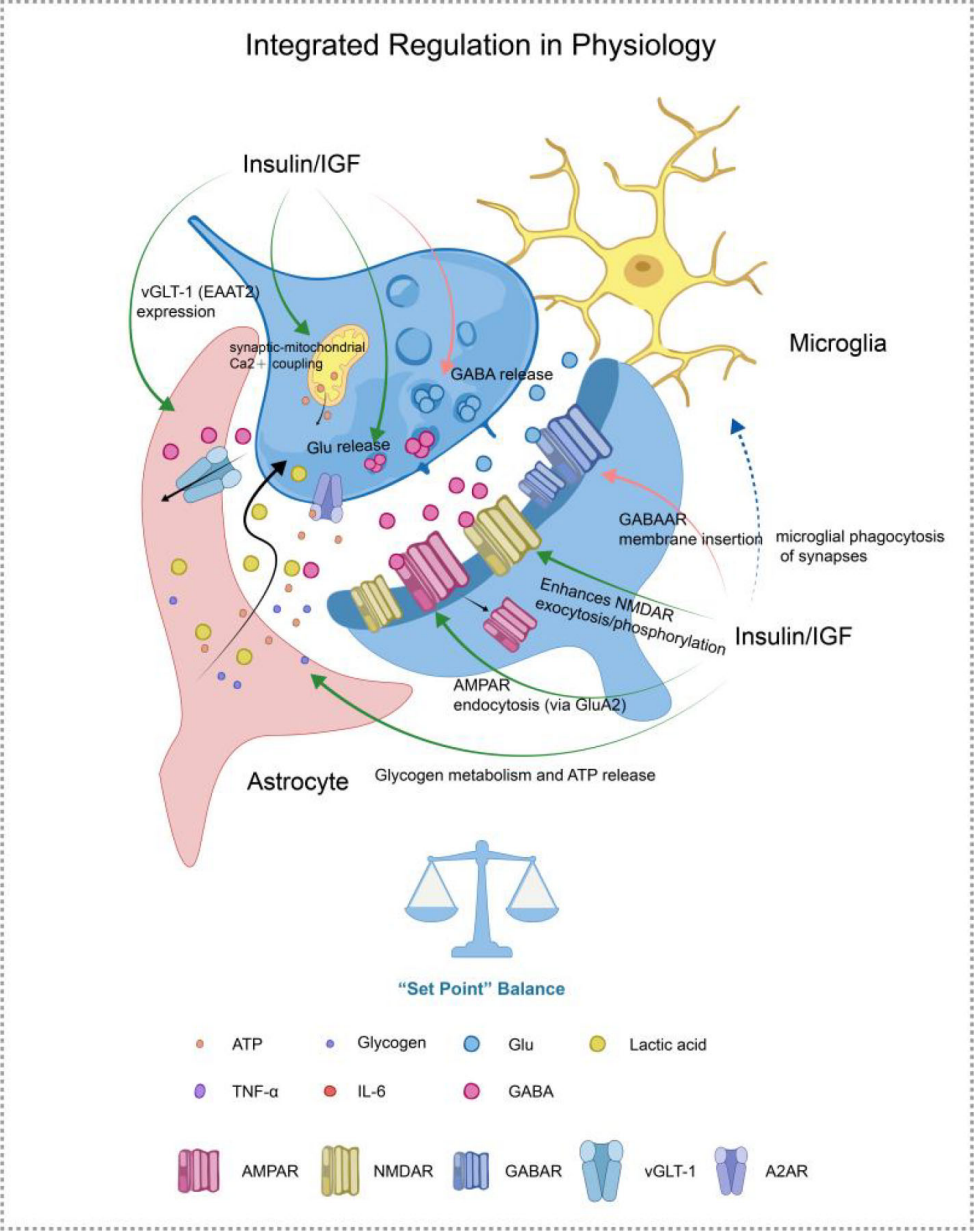
Insulin/IGF-1 signaling as a regulator of synaptic homeostasis. Insulin and IGF-1 signaling act directly on neurons by regulating receptor trafficking and presynaptic release probability. In parallel, they orchestrate astrocytic functions, including glutamate clearance via GLT-1, metabolic coupling through glycogenolysis and lactate shuttling, and modulation of purinergic signaling (ATP/adenosine). Together, these actions stabilize the excitatory–inhibitory balance and maintain the synaptic homeostatic “set-point”.

In conclusion, insulin and IGF-1 signaling maintain synaptic and network homeostasis under physiological conditions through integrated mechanisms such as synaptic scaling, metabolic coupling, and feedback regulation of firing rates. This multimodal regulatory capacity underscores their pivotal position in neural function. **Figure 2** summarizes the pivotal role of Insulin and IGF-1 signaling in maintaining synaptic homeostasis under physiological conditions.

Collectively, insulin and IGF signaling coordinate synaptic regulation across molecular, structural, metabolic, and microenvironmental dimensions. These multilevel mechanisms converge to stabilize circuit function under physiological conditions. For clarity, the principal regulatory axes discussed above are summarized in **Table 2**.

**Table 2:** Multilevel regulatory roles of insulin and IGF signaling in synaptic and circuit homeostasis.

Regulatory dimension	Key molecular targets	Functional outcome	Disease relevance
Synaptic Transmission	AMPAR (GluR1/2), NMDAR (NR2A/B), PKMζ	Modulation of synaptic strength;LTP/LTD threshold	Impaired LTP in DE, AD
Network Excitability & Homeostatic Scaling	AMPAR; BK/KATP channels; Mitochondrial MCUc	Activity downscaling, E/I balance	Network instability in DE
Structural Plasticity	Rac1, IRSp53, actin cytoskeleton	Dendritic spine formation and maintenance	Spine loss in AD, PD
Metabolic Coupling	Mitochondrial MCUc; GLUTs; GLT-1; IDE	Energy supply,	Synaptic vulnerability
Microenvironmental Regulation	GLT-1, microglial metabolism, HMGB1/TLR4	Glial support of synapses; neuroimmune modulation; glutamate clearance	Aβ accumulation, PD neuroinflammation
Developmental Circuit Wiring	IGF-2; SHANK3; mTOR	Circuit maturation	ASD-related dysfunction

## 5 Insulin and IGF signalling regulation of synaptic and neuronal circuits: implications for disease

DE represents a significant neurocognitive sequela of diabetes mellitus characterized by progressive synaptic dysfunction, neuronal loss, and brain atrophy that extends beyond vascular pathology (106). Its pathogenesis involves disruption of key neurotrophic signaling pathways, including insulin, IGF-1, and IGF-2. Insulin resistance attenuates PI3K/Akt signaling, increasing susceptibility to hyperglycemia-induced apoptosis and altering NMDA/AMPA receptor function, thereby impairing hippocampal synaptic plasticity and memory consolidation (107). Disrupted insulin signaling also dysregulates the potassium channel Kv1.3 in the olfactory bulb, modifying neuronal excitability and contributing to cognitive deficits (107).

Reduced IGF-1 receptor activation in the hippocampus and cortex compromises local paracrine/autocrine support of glutamatergic synapses, weakening long-term potentiation and learning (108). Loss of IGF-1–mediated mitochondrial protection further aggravates neuronal injury through ATP depletion and oxidative stress (106). In parallel, diminished IGF-2 signaling—partly due to reduced choroid plexus production—impairs neuroprotection and may alter amyloid-β clearance, facilitating protein aggregation (80).

Collectively, disruption of insulin, IGF-1, and IGF-2 signaling drives synaptic failure, mitochondrial dysfunction, apoptosis, and structural degeneration in DE, while simultaneously increasing vulnerability to neurodegenerative diseases, emotional disorders, and autism spectrum disorder, as discussed below.

### 5.1 Diabetic encephalopathy as a prototype of metabolic–synaptic uncoupling

Synaptic impairments are consistently observed in diabetic models, positioning diabetic encephalopathy as a prototypical condition in which disrupted insulin and IGF signaling destabilizes neuronal circuits. Central insulin resistance and IGF-1 deficiency lead to dendritic atrophy in CA3 and dentate gyrus neurons, reduced spine density in CA1, and downregulation of synaptic proteins including drebrin, synaptophysin, PSD-95, and MAP-2 (109–112). Excessive GSK3β activation and impaired PAK/LIMK/cofilin signaling disrupt actin dynamics and synaptogenesis, while reduced ILK activity diminishes AMPAR expression (109, 113).

Functionally, these structural deficits translate into impaired glutamatergic transmission, reflected by reduced mEPSC amplitude and frequency (111), suppressed CA3–CA1 transmission (114, 115), and impaired NMDAR-dependent LTP (116, 117). Molecular alterations include decreased phosphorylation of NR1 and reduced NR2A/NR2B and GluR1 expression, impaired presynaptic glutamate release (111, 115, 118–120), and diminished Na^+^/K^+^-ATPase activity (26, 121). IGF-1 deficiency further induces excitatory–inhibitory imbalance through astrocyte-dependent glutamate accumulation (122, 123). Behaviorally, these circuit-level abnormalities manifest as deficits in learning, memory, and exploratory activity.

Collectively, diabetic encephalopathy exemplifies how insulin/IGF signaling integrates metabolic status with synaptic protein turnover, receptor trafficking, cytoskeletal remodeling, and plasticity maintenance, thereby linking systemic metabolic dysfunction to network instability.

### 5.2 Extension to neurodegenerative disorders: synaptic homeostasis collapse in AD and PD

The mechanistic framework established in diabetic encephalopathy extends to neurodegenerative diseases characterized by synaptic vulnerability.

In Alzheimer’s disease, aberrant IRS-1 serine phosphorylation disrupts PI3K–Akt signaling, enhances GSK3β activity, and exacerbates tau hyperphosphorylation, destabilizing the synaptic cytoskeleton (124, 125). In an Aβ-rich environment, this leads to decreased synaptic protein expression and structural deterioration (124). Concurrently, dysregulation of the IGF2–IGF2R axis is associated with abnormalities in synaptic membrane protein trafficking and degradation, impairing spine formation and weakening synaptic stability (15, 124). Reduced insulin-degrading enzyme (IDE) limits Aβ clearance and further suppresses insulin receptor signaling, creating a vicious cycle (113, 126). Moreover, decreased phosphorylation of NMDA receptor subunits NR1 and NR2B—commonly observed in the AD hippocampus—is associated with reduced postsynaptic depolarization and impaired LTP (125, 127). Together, these changes reflect a breakdown of insulin and IGF-dependent synaptic homeostatic compensation.

Impairment of insulin and IGF-1 signaling, which regulates multiple neuroprotective processes, further accelerates dopaminergic neuron loss and synaptic deterioration (128, 129). In Parkinson’s disease, insulin resistance accelerates dopaminergic synaptic degeneration by suppressing Akt and activating stress-related MAPK pathways, reducing PSD-95, synaptophysin, and synapsin expression (130, 131). Disrupted insulin–dopamine coupling impairs TH activity, DAT expression, vesicle release, and D1/D2 pathway balance within basal ganglia circuits (132, 133). Conversely, IGF-1 activation of Akt–GSK3β signaling mitigates α-synuclein aggregation, enhances receptor trafficking, and restores dopaminergic firing patterns (41, 134–136). In midbrain organoid models, insulin resistance has been found to decrease dopaminergic neuron firing rate and burst activity, whereas IGF-1 supplementation restores both synaptic and metabolic function through activation of the Akt–mTOR pathway (131, 137, 138). Thus, in both AD and PD, metabolic signaling failure converges on synaptic and circuit destabilization rather than merely protein aggregation.

### 5.3 Neurodevelopmental and emotional disorders: plasticity imbalance and circuit dysregulation

In autism spectrum disorder (ASD), reduced IGF-1 signaling intersects with SHANK3-dependent postsynaptic scaffolding, influencing dendritic spine maturation and AMPA/NMDA receptor trafficking via the IGF–AKT–mTOR pathway (35, 139–141). Clinically, early controlled trials of IGF-1 in ASD-related genetic conditions demonstrated improvements in social behavior, repetitive behaviors, and sensory reactivity with acceptable safety profiles (142). The clinical relevance of IGF-related pathways is further underscored by the FDA approval of trofinetide, an IGF-1 analog, for Rett syndrome, and Orphan Drug designation of IGF-1 for Fragile X syndrome and SHANK3-related disorders (143).

In emotional disorders, conditional deletion of insulin or IGF-1 receptors in the hippocampus and central amygdala lowers GluA1 expression and induces anxiety-like behaviors (120). Similarly, insulin resistance suppresses glutamatergic vesicle release from basolateral amygdala to nucleus accumbens neurons, promoting depressive-like and social avoidance behaviors (144). Beyond glutamatergic modulation, central insulin resistance directly perturbs dopaminergic circuits involved in reward and motivation, thereby linking metabolic dysfunction to affective dysregulation (145). In contrast, IGF-1 signaling exerts a modulatory effect on excitatory transmission in prefrontal layer V pyramidal neurons; its deficiency disrupts projections to the nucleus accumbens and ventral tegmental area, exacerbating emotional instability (41). Akt–mTOR suppression limits protein synthesis (146, 147), while Akt–GSK3β dysregulation weakens receptor phosphorylation and synaptic strength (27), producing a state of “homeostatic drift” characterized by reduced excitability and plasticity. Unlike AD or PD, these alterations are largely functional and potentially reversible, highlighting insulin/IGF signaling as a dynamic regulator of circuit stability. Unlike AD or PD, these alterations are largely functional and potentially reversible, highlighting insulin/IGF signaling as a dynamic regulator of circuit stability.

## 6 Insulin and IGF signaling as a therapeutic target for diabetic encephalopathy

Brain insulin resistance is a central pathological mechanism that links systemic metabolic dysregulation, chronic neuroinflammation, and the failure of synaptic homeostasis. Molecular disruptions, including diminished PI3K/Akt signaling, aberrant GSK-3β activation, and mitochondrial dysfunction, converge to impair neuronal adaptability and network stability (148). This integrated pathophysiology establishes the restoration of central Insulin and IGF tone as a rational therapeutic cornerstone for counteracting cognitive decline in DE.

Therapeutic exploration has thus evolved from peripheral glucoregulation toward interventions that directly engage neural insulin pathways. Emerging strategies form a coherent hierarchy, from molecular reactivation to systemic support.

Direct enhancement of Insulin and IGF signaling represents the most straightforward approach to modifying synaptic function. Classical agents such as GLP-1 receptor agonists (e.g., liraglutide, semaglutide) cross the blood-brain barrier to activate PI3K/Akt and inhibit NF-κB, with clinical studies reporting cognitive benefits linked to improved synaptic health in diabetic patients (149, 150). Next-generation dual GLP-1/GIP agonists like tirzepatide demonstrate superior efficacy in preclinical models, rescuing hippocampal plasticity and memory by mitigating local insulin resistance and inflammation (151, 152). Similarly, IGF-2 supplementation reverses cognitive and synaptic deficits in models of developmental compromise, highlighting its specific neurotrophic role (82). Bioactive compounds such as walnut-derived peptides further exemplify this nodal strategy, and improving cognitive outcomes in T2DM mice via concurrent PI3K/Akt activation and NLRP3 suppression (153).

Modulation of the neuroinflammatory and metabolic milieu provides essential permissive conditions for signal restoration. Anti-inflammatory agents like avenanthramide-C and schisandrin A alleviate diabetes-associated cognitive impairment by targeting NOD1-driven inflammation and ferroptosis, respectively, thereby removing critical barriers to synaptic resilience (154, 155). The metabolic modulator medium-chain triglycerides (MCTs) supply alternative cerebral energy substrates, supporting fundamental neuronal bioenergetics and cognitive function in AD models (156). Even classical insulin sensitizers (e.g., pioglitazone), despite limited clinical success in AD trials, validate the principle of improving central insulin responsiveness via anti-inflammatory mechanisms (157).

Epigenetic and genetic regulatory layers offer potential for enduring modulation. Age-associated DNA hypermethylation of the hippocampal IGF-2 gene is linked to plasticity and memory decline, suggesting demethylation as a novel therapeutic avenue (158), suggesting that demethylation could be a novel avenue for reactivating endogenous trophic support for synapses. Furthermore, the ApoE genotype modulates hippocampal insulin sensitivity and cognition in a sex-dependent manner, underscoring the need for personalized therapeutic design (159).

This multiscale understanding is catalyzing the development of integrative pharmacologic agents. Compounds like tirzepatide and SEP-363856 exemplify this shift, simultaneously engaging metabolic, anti-inflammatory, and neurotrophic pathways to promote network stability (151, 152, 160). Translational success hinges on overcoming persistent challenges in drug delivery and precise neuromodulation. While intranasal insulin facilitates direct CNS delivery, its variable clinical efficacy highlights the complexity of achieving consistent synaptic modulation (161, 162). Advances in nanocarrier and exosome-based delivery systems promise improved brain targeting and pharmacokinetics (163). Safety considerations remain paramount, as evidenced by findings that excessive insulin can predispose to excitatory toxicity, a risk potentially mitgated by adjuncts like AAC2 (156).

In summary, the therapeutic landscape for diabetic encephalopathy has evolved into a multi-level, integrated restorative system aimed squarely at synaptic homeostasis. It begins with a deep understanding of the core Insulin and IGF signaling pathway and its pivotal role in governing synaptic strength, plasticity, and stability. This integrated logic has spurred the development of novel drugs capable of synergizing multiple mechanisms to recalibrate the synaptic “set-point.”

## 7 Concluding remarks

This review synthesizes current evidence indicating that Insulin and IGF signaling pathways serve as central, multi-tiered regulators of synaptic and network homeostasis within the central nervous system. Beyond their established metabolic functions, these pathways are crucial modulators of synaptic transmission, plasticity, structural integrity, and the synaptic microenvironment. Considering their critical role in maintaining synaptic and network homeostasis, insulin and IGF signaling pathways represent promising therapeutic targets. Strategies aiming to restore physiological signaling—such as intranasal insulin delivery, GLP-1 receptor agonists, IGF supplementation, or modulation of downstream effectors—offer rational approaches to ameliorate synaptic dysfunction and cognitive impairment. Future research should focus on elucidating the molecular switches that determine these pathways’ dual effects, facilitating the development of context-specific interventions for brain disorders involving metabolic and signaling dysregulation.
